# Imaging of GBM in the Age of Molecular Markers and MRI Guided Adaptive Radiation Therapy

**DOI:** 10.3390/jcm11195961

**Published:** 2022-10-10

**Authors:** Salah Dajani, Virginia B. Hill, John A. Kalapurakal, Craig M. Horbinski, Eric G. Nesbit, Sean Sachdev, Amulya Yalamanchili, Tarita O. Thomas

**Affiliations:** Neuroradiology Section, Department of Radiology, Northwestern University Feinberg School of Medicine, Chicago, IL 60611, USA

**Keywords:** GBM, MRI guided adaptive radiation therapy, MRI guided radiation therapy, imaging of GBM, Machine learning

## Abstract

Glioblastoma (GBM) continues to be one of the most lethal malignancies and is almost always fatal. In this review article, the role of radiation therapy, systemic therapy, as well as the molecular basis of classifying GBM is described. Technological advances in the treatment of GBM are outlined as well as the diagnostic imaging characteristics of this tumor. In addition, factors that affect prognosis such as differentiating progression from treatment effect is discussed. The role of MRI guided radiation therapy and how this technology may provide a mechanism to improve the care of patients with this disease are described.

## 1. Introduction

Glioblastoma (GBM) continues to be one of the most lethal malignancies and is almost always fatal. In the United States, there are approximately 75,000 new brain tumors diagnosed annually, with GBM being the most common primary malignant brain tumor [1]. Patients diagnosed with GBM commonly present with symptoms including headache, neurological deficit, seizure, nausea, vomiting, or other neurologic sequelae. Many patients have significant disease burden at initial presentation and prognosis is poor with a median survival under 2 years [2]. Even with significant technological advancements, practice-changing systemic therapies, and improved surgical techniques, there continue to be poor overall survival rates among patients with GBM, with only 10% of patients surviving up to 5 years [3].

The mainstay of initial diagnosis includes neurological clinical changes with corresponding diagnostic imaging changes in the brain suspicious for GBM. Whenever feasible, patients undergo craniotomy with the goal of maximal safe removal of the tumor and relief of mass effect. When craniotomy is contraindicated, stereotactic biopsy is performed instead. Despite maximal removal, significant disease burden remains as autopsies with clinicopathologic analysis have shown microscopic extension of up to 2.0 cm from the edge of the tumor [4]. After maximal safe resection, patients undergo 6 weeks of radiotherapy with concurrent temozolomide (TMZ) followed by maintenance TMZ as standard of care [3]. The addition of TMZ has shown a survival benefit, particularly for patients with O_6_-methylguanine DNA methyltransferase (*MGMT*) promoter methylated tumors, which is present in approximately 50% of patients [5]. The current standard of care includes the addition of antimitotic treatment known as tumor-treating fields to maintenance TMZ [6,7].

## 2. Role of Radiotherapy in GBM

Prior autopsy studies have underlined the infiltrative tendencies of GBM and have shown microscopic extension up to 2.0 cm from the border of the resection cavity, indicating that there continues to be a significant risk even after maximal safe resection [4]. External beam radiotherapy continues to be a mainstay of treatment to address this concern. A series of historical studies, the Brain Tumor Study Group protocols (BTSG 66-01, 69-01, 72-01), have established a dose-effect relationship and have shown that higher doses of adjuvant radiotherapy improve survival [8,9,10,11]. These three studies enrolled a total of 621 patients who had undergone resection with histologically confirmed GBM, prior to the molecular understanding of this disease. After resection, the patients received either no radiotherapy or adjuvant therapy to either ≤45.0 Gy, 50.0 Gy, 55.0 Gy, or 60.0 Gy given in doses of 171–200 cGy daily with five fractions per week. Median survival for these patients was 18.0 weeks, 13.5 weeks, 28.0 weeks, 36.0 weeks, and 42.0 weeks, respectively. These results showed that there was an improved median survival with dose escalation to 60 Gy [8]. Subsequently, multiple dose escalation studies including the most recent NRG trial BN001 have been performed but have thus far failed to show survival improvement with dose regimens above 60 Gy [12,13,14,15,16]. The current standard of care entails forming a clinical target volume (CTV) that includes the surgical resection cavity, residual contrast enhancing tissue, associated peritumoral hyperintensity based on T2-FLAIR signal, along with a 2.0 cm margin to cover for microscopic extension, and treating to 46 Gy. A 2 cm margin has been empirically determined, as over 80% of recurrences have been found within 2 cm of the contrast-enhanced lesion [17,18]. This initial treatment is then followed by a sequential boost of 14 Gy to a total dose of 60 Gy to treat a clinical target volume which includes only the resection cavity, residual contrast enhancing tissue, and a 2.0 cm margin. In summary, the 46 Gy field will cover the resection cavity plus regions of T2-FLAIR along with a 2.0 cm margin, while the 60 Gy field will omit regions of T2-FLAIR and target only the resection cavity with a 2.0 cm margin. This standard of care treatment protocol has been established based on NRG trial guidelines and the consensus of a panel of CNS radiation oncologists [19].

## 3. Role of Systemic Therapy

Historically, early trials of chemotherapy were negative, however, later meta-analysis showed a modest survival benefit with nitrosoureas [20]. The nitrosoureas are older chemotherapeutic agents that function as an alkylating agent, forming interstrand crosslinks in DNA, preventing replication and transcription. These agents were commonly combined with radiotherapy and still only provided a modest survival benefit with an absolute increased 1-year survival of 6% [20,21]. The current backbone of systemic therapy for GBM is TMZ, which is a more modern alkylating/methylating agent that targets the N-7 or O-6 positions of guanine residues in DNA. The role of TMZ was established in the EORTC 26981/22981 and NCIC CE.3 trial, also known as the Stupp trial, which compared post-operative radiotherapy alone to post-operative radiotherapy with concurrent TMZ followed by up to six cycles of maintenance TMZ. The TMZ arm had a significantly longer overall survival, 27.2% at 2 years and 10% at 5 years, compared to 10.9% and 1.9% in the radiotherapy alone arm, respectively [3]. Furthermore, compared to older systemic therapy regimens, TMZ was well tolerated with only 7% of patients experiencing grade 3 or 4 hematologic toxic effects [22]. In a companion publication to the EORTC 26981/22981 trial it was found that patients with *MGMT* promoter methylated tumors treated with TMZ derived an additional median survival benefit when compared to patients without *MGMT* promoter methylated tumors, with a median survival of 21.7 vs. 15.3 months, respectively [23]. Further evaluation has also confirmed the prognostic significance of *MGMT* promoter methylation status, showing that *MGMT* promoter methylated tumors experience a greater survival benefit with the current standard of care treatment [24].

In the setting of recurrence, it is exceptionally difficult to determine the best therapeutic regimen. If patients have *MGMT* promoter methylated tumors, and do not display disease progression throughout maintenance treatment with TMZ, and experience disease control for an adequate amount of time after completing maintenance TMZ, they can be re-challenged with TMZ [25]. Patients who do not meet these criteria are typically treated with other agents such as nitrosoureas or bevacizumab. These patients tend to do poorly with a median survival of under one year [26,27].

These abysmal findings demonstrate the need for novel therapeutic interventions in both the *MGMT* promoter unmethylated population and in patients with disease recurrence.

## 4. Molecular Basis of GBM

Prior to the World Health Organization (WHO) Classification of Tumors of the Central Nervous System (CNS) fourth edition, molecular characteristics were not a major factor in the classification of tumors as histological features dictated tumor grading. In 2016, when the fourth edition was released, molecular characteristics were integrated into the classification system. Previously, histological features were sufficient to deem a tumor as grade 4 and classify it as a GBM, regardless of isocitrate dehydrogenase (*IDH*) status. With the new 2021 classification system (WHO fifth edition), *TERT* promoter mutation, *EGFR* amplification, and +7/−10 copy number changes in IDH-wildtype diffuse astrocytomas are awarded a glioblastoma, IDH-wildtype CNS WHO grade 4 designation despite harboring histological features that would previously be rated as a lower grade. This change in classification criterion was made to adequately group patients with the poorest prognosis [28].

Several other molecular biomarkers of prognostic significance have been identified. Both the telomerase reverse transcriptase (*TERT*) promoter and epidermal growth factor receptor (*EGFR*) have been shown to have a prognostic significance among patients with GBM. *TERT* promoter mutation vs. *TERT* promoter wild type has been found to be associated with an older age (median 59.6 vs. 53.6 years), and with a poorer prognosis in *IDH* wild type GBMs with an overall survival of 13.7 vs. 17.5 months. Additionally, there appears to be an interplay between *TERT* and *EGFR*. Patients with a *TERT* promoter wild type and *EGFR* wild type tumor had approximately double the overall survival of patients harboring a *TERT* promoter wild type tumor with *EGFR* alteration, with an overall survival of 26.6 vs. 13.3 months. To further complicate this, in *EGFR* altered tumors, *TERT* promoter mutation was associated with a longer survival than *TERT* promoter wild type. Finally, patients harboring neither mutation had the best prognosis [29].

The Cancer Genome Atlas (TCGA) has also examined molecular characterics of GBM and has shown that multiple genes (*ERBB2*, *NF1*, *TP53*, *PIK3R1*, *PDGFRA/IDH1*, etc.) and pathways possess aberrations that may be clinically relevant [30,31]. Molecular analysis of transcription levels, genetic alterations, and DNA methylation has also allowed for further subtyping of GBM into 4 classes, proneural, neural, classical, and mesenchymal [31,32,33]. Response to treatment differs among subtypes with the classical subtype receiving the greatest benefit from treatment and the proneural subtype showing little to no benefit [31]. Additionally, *MGMT* promoter methylation status may serve as a predictive biomarker to treatment response in the classical subtype and not the proneural subtype [34]. These findings reinforce the notion that molecular testing plays a critical role in elucidating prognosis as there is a complicated interplay between multiple genes and pathways.

With over 90% of GBM patients experiencing recurrence, tumor recurrence continues to be a leading cause of mortality with limited treatment options [35,36]. Tumor recurrences display distinct genomic alterations when compared to the initial tumor. Molecular analysis shows that driver mutations of the initial tumor are undetected in some recurrences such as driver mutations in *TP53*, *ATRX*, *SMARCA4*, and *BRAF*. Tumors initially treated with TMZ were also found to be hypermutated at recurrence and harbored *RB* and *Akt-mTOR* mutations known to be a sign of TMZ-induced mutagenesis [37]. Given the heterogeneity in the histological patterns and genomic alterations of GBM and GBM recurrence, further molecular classification is necessary to appropriately develop personalized treatment.

## 5. Types of Radiotherapy Devices for GBM

The role of radiotherapy as a cornerstone of treatment for GBM after surgical resection has been well established. There are multiple techniques available to deliver radiation that have been explored over the years.

LINAC—The most common technique utilized is external beam radiotherapy through the use of a linear accelerator (LINAC). LINAC based treatments provide photon radiation. Typical LINACs have on-board imaging capabilities which allow verification with a cone beam CT or kV X-ray image. The on-board imaging is utilized for confirming patient positioning and verifying treatment field shape, is not of diagnostic quality, is low resolution, and is not ideal for visualizing soft tissue such as brain. However, LINAC based treatment remains the standard of care at this time.

IORT—Intraoperative radiotherapy (IORT) is a method of radiotherapy that delivers a large single dose of radiation directly to the tumor tissue and resection cavity. In several small and limited studies IORT has been utilized to provide dose escalation, delivering 12–20 Gy intraoperatively with patients subsequently receiving adjuvant radiotherapy as well. The most recent studies in the TMZ era have been relatively small, consisting of approximately 15–50 patients. While these studies have shown manageable rates of toxicity and verified the safety of IORT, they have failed to show meaningful improvements in patient outcomes such as PFS or OS improvements [38,39]. As such, IORT is not a considered a standard of care practice for patients with GBM.

GammaTile—GammaTile brachytherapy (GT) is a bioresorbable 3D-collagen tile embedded with Cesium-131 sources which is implanted in the final stages of tumor resection. GT is a relatively new method to treat recurrent brain tumors. A recent study in *IDH* wildtype GBM treated with maximal safe resection and GT at the time of recurrence demonstrated an overall survival of 20 months for *MGMT* promoter unmethylated patients and 37 months for *MGMT* promoter methylated patients. GT safety profile is comparable to patients undergoing repeat surgery without GammaTile [40].

SRS—Stereotactic radiosurgery (SRS) is another method of radiotherapy that entails high dose per fraction irradiation. SRS may be performed with a LINAC or a cobalt source platform that utilizes multiple beams that converge in three dimensions on a small target. Through this technique, a much higher dose may be delivered, which leads to a more ablative treatment, causing higher rates of cell kill and significant damage to tumor vasculature [41,42]. Since a much higher dose is being utilized, it is critical to ensure accurate target localization and tight margins. Due to this, greater spatial resolution is required; therefore, it is recommended that magnetic resonance imaging is co-registered to aid in target volume delineation [43].

At present, stereotactic radiosurgery is commonly utilized in the treatment of brain metastases. There have been multiple prior attempts to establish a role for SRS in the treatment of GBMs, such as in the Radiation Therapy Oncology Group (RTOG) 9305 trial which compared postoperative resection cavity SRS plus external beam radiotherapy vs. resection plus external beam radiotherapy. Unfortunately, there were no significant survival differences or pattern of failure differences between the two arms [44]. As such, the role of SRS for the treatment of GBMs remains controversial and limited. Currently, SRS is most commonly utilized in the setting of recurrent GBM for patients with a high-performance status, a good response to the initial chemoradiotherapy treatment, a prolonged interval to recurrence, and limited volume disease [45].

Protons—Charged particle therapy, particularly proton therapy, has been gaining popularity in the last decade. Proton based radiation imparts a dosimetric benefit, allowing for a more favorable dose distribution, which in turn may reduce patient morbidity. A retrospective analysis comparing photon and proton treatment plans showed that proton therapy provided a dosimetric advantage by reducing dose to organs at risk [46]. Although proton therapy provides a more favorable dosimetry, it is unclear if this translates into improved clinical outcomes or reduced late toxicity. A recent prospective phase II randomized controlled trial compared proton therapy vs. intensity modulated radiotherapy for patients with newly diagnosed GBMs and utilized first cognitive failure as a primary endpoint. Despite the more favorable dosimetry, there were no significant differences in cognitive failure between the treatment arms. Additionally, there were no significant differences in progression free survival or overall survival [47]. Pending results from the NRG BN001 trial [Clinical Trial NCT02179086] will also allow a comparison between proton- and photon-based radiotherapy in the treatment of GBM. Thus far, proton therapy has not yet demonstrated improved treatment efficacy nor reduction in toxicity in the treatment of GBM.

MRI guided machine—Soft tissue imaging is critical for successful volume definition in GBM. As already mentioned, for GBM treatment it is necessary to co-register MRI scans to the radiotherapy planning scan in order to have the necessary spatial resolution to properly define the target volume and organs at risk. Furthermore, as image guided radiotherapy has become a mainstay of treatment, there has been a need for better soft tissue image guidance. This has led to the development of MRI guided radiotherapy machines.

## 6. Imaging Characteristics of GBM on MRI and PET

**Figure 1:**
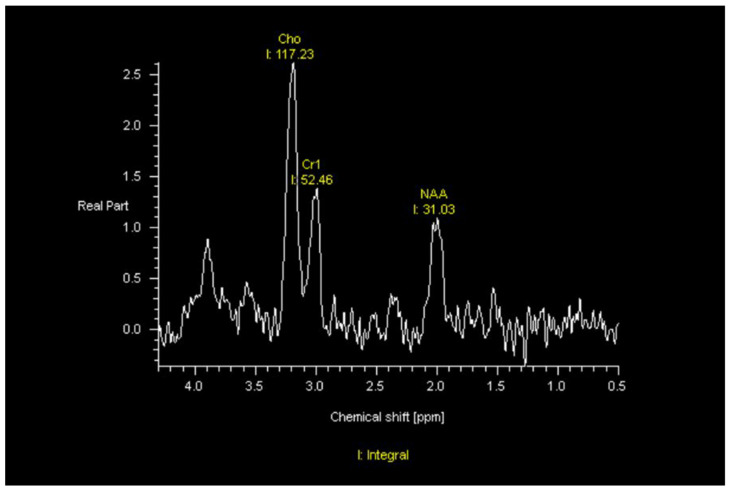
MR Spectroscopy shows reversal of Hunter’s angle with elevated choline (Cho), decreased creatine (Cr), and decreased N-acetyl-aspartase (NAA) in a patient with GBM status post radiation therapy, temozolomide, and tumor treating fields.

MRI—On MRI, GBMs typically demonstrate contrast-enhanced T1-weighted heterogeneous enhancement and irregular borders, central necrosis, and significant surrounding T2/FLAIR hyperintensity. The increased FLAIR signal in the peritumoral habitat reflects not only edema but also nonenhancing infiltrative tumor. The increased cellularity of these tumors often appears heterogeneously relatively T2-hypointense and may be associated with restricted diffusion. However, a minority of GBMs will manifest as ill-defined nonenhancing, T2/FLAIR hyperintense masses that are difficult to differentiate from low-grade gliomas or as smaller homogeneously enhancing well-defined masses that can be mistaken for metastases. GBMs at the corpus callosum can be difficult to differentiate from lymphoma. Tumefactive multiple sclerosis, infection, and subacute infarcts also can be difficult to differentiate from GBMs or other neoplasms. GBMs typically result in significant mass effect. GBMs can demonstrate increased corrected relative cerebral blood volume (rCBV) on dynamic susceptibility contrast (DSC) MR perfusion, increased permeability on dynamic contrast-enhanced (DCE) MR perfusion, and an abnormal tumor spectrum on MR spectroscopy, with reversal of Hunter’s angle—meaning increased choline (Cho), decreased creatine (Cr) and decreased N-acetyl-aspartase (NAA) (Figure 1).

PET—While PET scans performed with fluorodeoxyglucose (FDG) are not effective at delineating GBMs because the normal brain parenchyma takes up nearly as much of the FDG radiotracer as many tumors, other radiopharmaceuticals are more effective at demonstrating functional markers of malignancy. PET radiopharmaceuticals can be analogs of glucose (FDG) or nucleosides (18F-fluorothymidine [18F-FLT]), or can be amino acids labeled with a radioactive element detectable by imaging. Amino acids have demonstrated great success in GBM imaging. The amino acids are incorporated into proteins such as cell membrane transporters and, unlike contrast-enhanced MRI, do not depend upon the leakiness of the blood–brain barrier to be detected on imaging. For example, O-(2-[18F]fluoroethyl)-L-tyrosine (18F-FET) has been useful in targeting the highest-yield focus of the tumor for biopsy, delineating the margins of the tumor more accurately than DSC MR perfusion or 5-aminolevulinic acid (5-ALA), and demonstrating residual tumor volume more accurately than MRI [48]. However, mimics of tumors such as ischemic infarct, infection, and tumefactive demyelination can also demonstrate FET-PET positivity [48,49]. While 18F-fluorodihydroxyphenylalanine (18F-FDOPA) is not as broadly useful as FET-PET, it has the advantage of not requiring a cyclotron to produce it [48]. 18F-fluorothymidine [18F-FLT] is an analog of thymidine and is a marker of cellular proliferation. It has been used to differentiate between higher and lower grade gliomas, but has not been successful in differentiating low-grade gliomas from non-neoplastic processes. FLT has been helpful in predicting treatment success and overall survival after treatment with bevacizumab, an advantage over MRI since repair of the blood–brain barrier with bevacizumab treatment typically dramatically reduces edema, mass effect and enhancement on MRI but without a concomitant increase in survival [48]. In addition, carbon-11 methionine (11C-Methionine) has been helpful in differentiating tumor, with an excellent tumor-to-background ratio, but standardized uptake value (SUV) is not as helpful in glioblastomas due to their heterogeneous biology. However, newer parameters such as metabolic tumor volume and total lesion methionine metabolism have recently been shown to be helpful in predicting overall survival [50].

## 7. Radiomic and Radiogenomic Differentiation of Molecular Markers, Sex Differences, and Morphologic Subtypes of GBMs

Radiomics is the quantitative analysis of radiologic images, using statistics or machine learning/artificial intelligence, to correlate features on a large scale to underlying biologic processes or diagnoses. Radiogenomics is the correlation of these imaging features with gene expression patterns and mutations.

*IDH* wild type—Before the patient’s treatment plan can be formed, we must know whether the tumor is *IDH* wild type or mutated. Many groups have developed a variety of machine learning tools to predict the *IDH* status noninvasively, but recent deep learning models using convolutional neural networks have achieved high accuracy, sensitivity, specificity, and Dice scores (Dice coefficient indicates how closely a segmentation matches the ground truth) in automatically color coding and predicting *IDH* mutation status by analyzing only T2-weighted MRI—without contrast-enhanced MRI sequences—apparently obviating the administration of contrast material [51,52]. Yogananda et al. developed an algorithm that predicts *IDH* status and automatically segments and color codes the *IDH* wild type and mutated tumor segments on only T2-weighted images with an accuracy of 97.14%, sensitivity of 97%, specificity of 98%, area under the curve of 98% and Dice score of 85% [52].

*MGMT* promoter methylation—Machine learning models have been able to noninvasively detect *MGMT* promoter methylation on MRI analysis, with varying degrees of success. Recently, the same model described above that demonstrated high accuracy in detecting *IDH* mutation status also was used to detect and segment the *MGMT* promoter methylated tumor on MRI with an accuracy of 94.73%, sensitivity of 96.31%, specificity of 91.66%, and mean AUC of 93%. The Dice score for segmenting the tumor on MRI was 82%. This model again was successful using only T2-weighted images, possibly eliminating the need to administer contrast material [53].

*EGFR* alteration—One of the best-known drivers of aggressive behavior in GBMs is *EGFR* alteration, in specific, *EGFRvIII* mutation. Akbari et al. showed that multiparametric machine learning modeling could be used to detect *EGFRvIII* alteration with 85.3% accuracy, 86.3% specificity, 83.3% sensitivity, 85% AUC [54]. The spatial heterogeneity of the tumor in its entirety was particularly useful in stratifying patients for targeted therapy and for potentially monitoring changes during treatment. In addition, the machine learning model elucidated underlying biological processes that allow this genetic alteration to drive GBM’s aggressive behavior. For example, *EGFRvIII* alterations demonstrated enhancing tumor higher rCBV and lower ADC, revealing increased neovascularization and hypercellularity; small internal areas of low ADC, low vascularization and low water content within enhancing tumor, revealing hypercellularity and prenecrotic areas of the tumor; and nonenhancing areas with higher rCBV, lower ADC, and lower water content, revealing increased neovascularization and hypercellularity [54].

**Figure 2:**
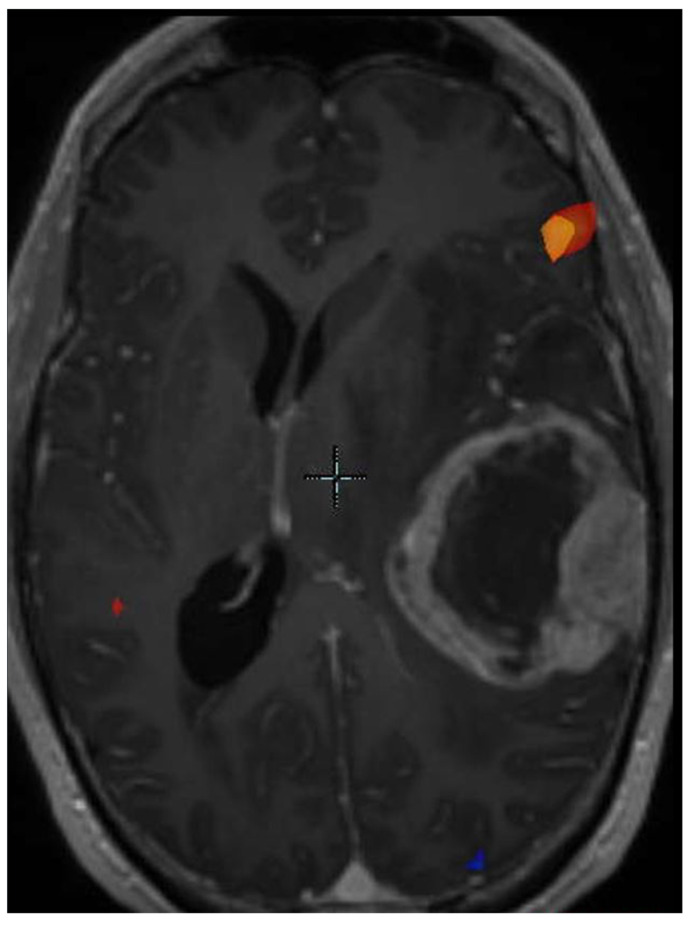
Functional MRI BOLD map superimposed on an MRI of an epithelioid GBM. Note the large cystic component.

*BRAF-V600E* Mutation—While the cases of *BRAF-V600E* mutated GBMs are too few for studies to demonstrate statistical significance and power, the potential for increased survival with targeted treatment merits discussion of the radiologic features of *BRAF-V600E-*mutated tumors. *BRAF-V600E* mutation, associated with epithelioid GBM, can be targeted by BRAF and MEK inhibitors such as dabrafenib and trametinib, which can lead to longer survival in some cases. However, some epithelioid GBMs result in shorter survival despite treatment, and investigation of additional molecular markers such as *TERT* promoter mutation and *CDKN2A/B* homozygous deletion is needed to predict which tumors will progress despite targeted therapy. Ishi et al. determined that *BRAF V600E*-mutated tumors usually manifested as a single contrast-enhancing cystic and solid lesion, contained a solid component involving the cortex, typically had large cysts with thin walls, were well-circumscribed with mild surrounding FLAIR signal, and sometimes demonstrated internal hemorrhage. These finding neared statistical significance [55]. Natsumeda et al. demonstrated similar findings, with 71% of cases demonstrating a single cyst, with a solid component involving the cortex in 75% of cases, and with a large cyst with thin walls (defined as at least 50% of the tumor size) in 50% of cases. Ninety-two percent of cases were well-circumscribed and 58% of cases had mild surrounding FLAIR signal [56]. In addition, some of the masses with hemorrhage had started as FLAIR lesions detected multiple years earlier. *BRAF-V600E*-mutated tumors tended to occur in patients younger than 55 years [56]. Some *BRAF V600E*-mutated tumors involve the dura and demonstrate a dural tail. Some arise in the frontal lobe, but most originate in the temporal lobe. A temporal lobe cystic and solid enhancing tumor (Figure 2) in a patient younger than 55 years should prompt next-generation sequencing of the tumor for *BRAF* mutation, in case targeted therapy may be initiated.

Differences in GBMs between Men and Women—Multiple studies have explored sex differences in molecular markers, survival, and signaling pathways between men and women with GBM. Beig et al. correlated the results of single sample gene set enrichment analysis (ssGSEA) with a radiomic risk score that included radiomic features on texture analysis from the FLAIR hyperintense, enhancing tumor and necrotic core segments of GBMs on MRIs [57]. In men, they found Laws energy features including spots and ripples in the enhancing tumor and peritumoral FLAIR hyperintense region were correlated with increased angiogenesis (with ripples likely representing abnormal vasculature) and cell adhesion. Increased Gabor wavelet features (possibly capturing hypercellularity and pseudopalisading cells) around and in the necrotic core also were correlated with cell adhesion and cell migration levels in the high risk male group with poor overall survival. In women, they found Laws energy edge features in the necrotic core correlated with immunologic signaling pathways, and Haralick inverse difference moment in the peritumoral FLAIR hyperintensity (suggesting increased homogeneity) was positively correlated with immunologic processes in the low-risk female group. The homogeneity of peritumoral FLAIR hyperintensity suggests lack of infiltrating nonenhancing tumor intermixed with edema in this region. Immune-function genes on the X chromosome may help explain this, possibly inhibiting IL-6 signaling in the low-risk female group with reduced activation of regulatory T cells and lower levels of tumor-associated macrophages.

Morphologic Subtypes of GBM—Building upon earlier works such as the 2014 article by Gevaert et al. that showed the potential for radiogenomic MRI studies to predict molecular markers and survival, Choi et al. used a machine learning algorithm to identify three morphologic subtypes of GBM with differing survival characteristics [58,59]. They showed a correlation between imaging subtype, genomics (using ssGSEA), and underlying biological processes—proving that radiomics can be used as a biomarker. The first subtype comprised solid masses with necrosis and heterogeneous enhancement and demonstrated the worst prognosis (underlying increased lysosomal activity and increased autophagy). The second subtype of rim-enhancing necrotic tumors was associated with a spherical shape with internal homogeneous T2 hyperintensity and had an intermediate prognosis (increased chemotaxis and an increased pro-inflammatory response). The third subtype was cystic appearing and had the most favorable response (downregulation of the MAPK pathway). Of note, the increased autophagy seen in the first heterogeneously enhancing subtype has been correlated with resistance to TMZ. In the rim-enhancing spherical subtype, the prevalence of necrosis correlates with inflammation in the surrounding parenchyma. The TNF-alpha pathway was upregulated in these tumors.

## 8. Progression of Disease versus Treatment Effect

After a GBM has been treated, it can be impossible on morphologic MRI (i.e., routine MRI, without advanced imaging techniques such as MR perfusion) to tell the difference between recurrent tumor and treatment effect. Treatment effect includes pseudoprogression and radiation necrosis. It is important to remember that enhancement on an MRI of the brain simply means disturbance of the blood–brain barrier by any mechanism. It is also important to remember that because of the inflammation related to radiation therapy, enhancement on MRI earlier than one month after radiation therapy is predictive of neither disease progression nor treatment-related changes.

Brain tissue resected after glioma therapy that has apparent glioma cells within it, via pathologic examination, is most often diagnosed as “recurrent/residual glioma.” This is because it is often very difficult to confidently determine, based solely on microscopic morphology, whether the glioma cells that are present within a specimen were there prior to adjuvant therapy or regrew after such therapy. To date, there are no widely accepted pathologic criteria for what should count as “recurrent” versus “residual.” However, it seems intuitive that large regions of tissue with healthy-appearing, mitotically active glioma cells should be considered recurrent, whereas tissue with mostly treatment-related necrosis and only scattered glioma cells with obvious damage (e.g., extreme nuclear atypia and extensive cytoplasmic vacuolation) would suggest only residual glioma.

On the subject of differentiating treatment-related necrosis from the necrosis that happens in grade 4 astrocytomas and GBMs, or grade 3 oligodendrogliomas, the former shows widespread indiscriminate tissue destruction involving nonneoplastic elements like blood vessels, whereas the latter is more focused and mostly contains dead tumor cells.

Progression of Disease—Previously, imaging evidence of progression of viable tumor defined in the MacDonald criteria took only the enhancing tumor into account [60]. This system did not acknowledge the importance of the infiltrative nonenhancing FLAIR hyperintense tumor that may surround an enhancing component, or the need for steroids or the clinical status of the patient. The Revised Assessment in Neuro-oncology (RANO) Criteria is more accurate than the McDonald criteria because it includes not only the growth of a pre-existing enhancing component, but also the growth of T2/FLAIR signal, the presence of any new lesions, dependence on corticosteroids, and the patient’s clinical status [61]. Modified RANO criteria, including a volumetric tumor measurement, were published in 2017 and are summarized in Table 1 [62,63]. Immunotherapy Response Assessment in Neuro-Oncology (iRANO) was published in 2015 to address the confusing imaging appearance on follow-up MRI in patients receiving immunotherapy [64]. In iRANO, because new lesions could represent inflammatory responses to immunotherapy rather than progressive disease, if the duration of immunotherapy is less than or equal to 6 months and progressive disease is suspected, therapy is continued for 3 additional months unless there is clinical worsening. After 3 months, a repeat scan is performed and serves as the determination of whether the initial scan showing concerning findings represented pseudoprogression or true progression. The determination is back-dated to that scan. If however a patient demonstrates clinical decline, that is considered to be progressive disease.

Pseudoprogression—The term “pseudoprogression” refers to the subacute increase in size of an enhancing and/or nonenhancing lesion within the radiation portal that stabilizes or resolves on its own without a change in the patient’s treatment. Pseudoprogression is thus a radiologic and clinical diagnosis, while radiation necrosis is a histologic definition. Pseudoprogression occurs in a variable percentage of patients with GBMs undergoing treatment, ranging from 2–50% in the literature [65]. It is more likely to occur in tumors with *MGMT* promoter methylation and is associated with increased overall survival [65,66]. In Brandes et al.’s study, median survival was 43.6 months in patients with *MGMT*-promoter methylated tumors but only 16.8 months in patients with unmethylated tumors [66]. Similarly, median survival was 38 months in patients with pseudoprogression, but only 20.2 months in patients with neither progression of disease nor pseudoprogression and 10.2 months in patients with early disease progression. Thus, pseudoprogression is associated with an improved prognosis. Pseudoprogression usually occurs within the first 3 months after the completion of radiation therapy, but has been reported as late as 10 months after radiation therapy. While its physiological underpinnings are not clearly agreed upon, it is thought to relate to post-treatment inflammation and increased capillary permeability associated with vascular hyalinzation with or without fibrinoid necrosis, thrombotic-type tumor necrosis as well as oligodendroglial damage associated with demyelination, reactive gliosis, edema, and damage to astrocytic foot processes causing disruption of the blood–brain barrier [67,68,69]. Enhancement, reflecting blood-brain-barrier breakdown, also may relate in part to the effect of cytokines released from cells during treatment-induced cellular hypoxia [70]. Other treatment-related insults that may contribute to enhancement not due to viable tumor include treatment-related necrosis, seizure-related changes, post-operative infarcts, and vascular changes related to decreasing steroid dose.

Radiation Necrosis—MRI changes due to radiation injury can be acute, subacute, or late. Changes that occur during radiation therapy (acute) or within the first 3 months after radiation therapy (subacute), which can be indistinguishable from pseudoprogression (see above), reflect injury to the tumor’s vasculature (vasodilation and increased permeability) and the blood–brain barrier. Months to years after radiation therapy (late changes), vessel damage also may result in necrosis and edema. While the mechanisms and outcomes of pseudoprogression and radiation necrosis are different, the histologic appearance is similar. Radiation necrosis is often symptomatic, requiring therapy.

SMART syndrome, which stands for Stroke-Like Migraine Attacks after Radiation Therapy, represents an additional late radiation injury. This syndrome can occur many years after radiation therapy to the brain and is associated with transient usually unilateral cortical imaging changes (however, imaging findings depend on the radiation fields) generally with sparing of the white matter. Possible MRI manifestations of this syndrome include unilateral cortical T2/FLAIR hyperintensity and enhancement, as well as restricted diffusion, susceptibility artifact in the subcortical white matter adjacent to the involved cortex, increased relative cerebral blood volume on dynamic-susceptibility-contrast MR perfusion, a white matter cavernous malformation or microhemorrhage remote from the acute abnormality, and subcortical edema [71,72,73,74]. In a study by Ota et al., young age was associated with a likelihood of complete recovery from SMART syndrome [71]. Steroid treatment at the time of diagnosis, subcortical susceptibility artifact adjacent to the cortical lesion, restricted diffusion, and subcortical edema were associated with a likelihood of a worse outcome. Of interest, increased rCBV, cortical enhancement and edema, and restricted diffusion nearly always resolved in SMART syndrome while subcortical susceptibility and subcortical edema often did not resolve [71].

**Figure 3:**
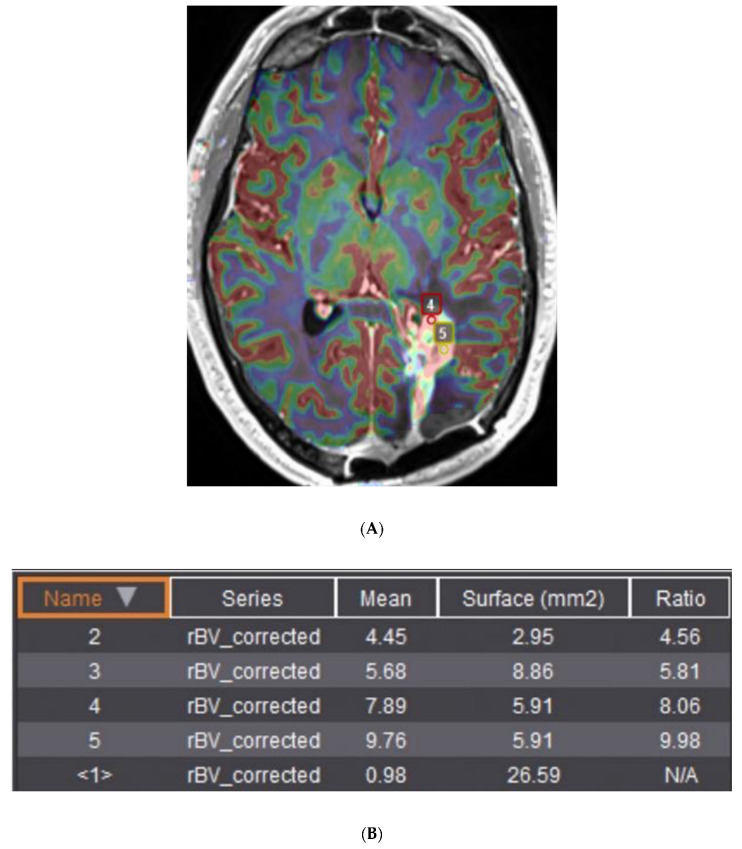
(**A**) Relative cerebral blood volume map superimposed on a gadolinium-enhanced MPRAGE image in a patient with recurrent GBM in the periatrial white matter and occipital lobe. (**B**) Ratios of corrected relative cerebral blood volume (2–5) relative to normal appearing white matter (<1>) are elevated, suggesting recurrent viable tumor rather than treatment effect. Points 1–3 not shown for brevity.

Differentiation between viable tumor and treatment-related changes on MRI.–Classically, DSC perfusion is used to differentiate between viable tumor and treatment effect with a sensitivity and specificity of 80–90% [75]. Elevated corrected rCBV (crCBV) is associated with viable tumor (with variability of the threshold in the literature and by site—in the original studies, a cutoff of 1.7, but in recent practice often a cutoff of 2 is used, and in some cases a cutoff of 3), while decreased crCBV is presumed to reflect treatment change (Figure 3). It is useful to follow crCBV over time, with an increase in CBV raising some concern for progressive disease and a decrease possibly reflecting treatment effect. Unfortunately, break-down of the blood–brain barrier such as with convection-aided chemotherapy delivery techniques and inflammatory responses related to immunotherapy have unpredictable effects on perfusion imaging—so much so that immunotherapy RANO (iRANO) waits until after 3–6 months of imaging and then backdates the determination of treatment effect versus recurrent tumor to the time of original change in the imaging appearance.

PET has been very successful in differentiating between treatment effect and progressive disease. For example, FET-PET is effective at differentiating between tumor progression and treatment effect [48]. While FDOPA-PET is not as broadly useful as FET-PET, it has been very useful in differentiating between treatment effect and recurrent GBM and has the advantage of not requiring a cyclotron to produce it [48].

Machine learning shows great promise for being able to noninvasively, accurately differentiate between treatment effect and tumor recurrence, with the achievable goal of obviating biopsy—especially in conjunction with traditional radiologist reads. Patel et al. combined clinical characteristics, genomic data and 307 quantitative imaging features to predict early GBM progression versus pseudoprogression with an area under the curve of 80% [76]. Tiwari et al. used texture analysis with a support vector machine to achieve 75% accuracy in differentiating between tumor and treatment effect, which increased to 92% when a consensus between the radiomic model and the expert readers was used [77]. Jang et al. used deep learning with a convolutional neural network (CNN) and long short-term memory (LSTM) techniques to predict pseudoprogression versus true progression with an area under the precision-recall curve of 86% [78]. Jang et al.’s model used imaging data, clinical data, and genomic data to predict viable tumor versus treatment effect with an attractive and easy-to-use user interface with a probability dial of progressive disease. Ismail et al. used shape features to differentiate true progression from pseudoprogression (accuracy 90.2%), with viable tumor demonstrating a compact round shape and pseudoprogression demonstrating an elliptic, elongated shape, both in the contrast-enhancing portion and the peritumoral FLAIR hyperintense region [79].

## 9. MRI Guided Machines

Imaging component—Magnetic resonance imaging linear accelerators (MRLINAC) provide improved soft tissue imaging, allowing for superior image guidance and adaptive treatment planning. Combining an MRI system and LINAC is an arduous task with numerous logistical obstacles. Firstly, the MRI imaging system relies on a high-powered magnet, which produces a magnetic field that may hamper the function of the multi-leaf collimator (MLC) of the LINAC [80]. Additionally, a magnetic field may influence the motion of electrons as they travel through the accelerating waveguide [81]. Furthermore, the presence of a LINAC near an MRI system may lead to interaction between the treatment beam and RF receiver coil, causing image quality degradation [82]. There are several different methods of overcoming these obstacles.

Currently there are two commercial systems available, with more to come in the future. The Elekta Unity utilizes a 1.5 T magnet, which is the highest-powered magnet commercially available in today’s MRLINACs. To overcome interference from this magnet the photon beam is placed on a rotating gantry which passes a superconducting cryostat configured to avoid angles that will interfere with imaging quality. Additionally, the electron gun is housed in a zero-field zone and utilizes specific shim settings to minimize image degradation [83].

The ViewRay MRIdian system utilizes similar tactics to avoid interference. Additionally, it utilizes a lower powered magnet at 0.35 T. This allows for sufficient image quality for image guidance and treatment adaption, however, it leads to a weaker magnetic field which places less stress on the LINAC [83]. The MRIdian also utilizes a balanced steady-state free precession sequence known as true fast imaging with steady state precession (TrueFISP) [84]. In TrueFISP the echo time and repetition time are reduced. This provides a mixture of T2/T1 weighted contrast, with a more predominant T2-weighted appearance, and allows for timely image acquisition [85,86].

Treatment planning systems are fully integrated into both the Unity and MRIdian, allowing for on-table treatment plan adaptive radiotherapy. This allows treatment to be re-planned in the event of organ motion or with target volume alteration during the course of treatment. In these systems, each daily scan is registered to the primary planning image along with the initial target volumes and organ at risk contours. These volumes can then be adjusted as appropriate. The original treatment plan is subsequently recalculated with an updated Monte Carlo dose calculation that is calibrated to account for the magnetic field as well. The software will provide a dose volume histogram (DVH) comparison of both plans. The user can then determine which plan is preferred, providing the patient with superior target coverage and sparing of organs at risk [84,87].

## 10. Role of MRI Linac in Assessing Tumor Response during Treatment

**Figure 4:**
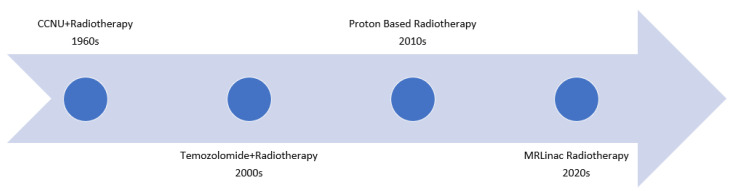
As far back as the 1960s and 1970s there have been clinical trials examining the role of systemic therapy combined with radiotherapy. Notably, the Walker Series first examined combining CCNU and radiotherapy in the Brain Tumor Study Group protocols BTSG 66-01, 69-01, 72-01. More recently, the Stupp trial changed the standard of care to include Temozolomide concurrently with radiotherapy. As technological advancements have been made, there has also been an interest in the potential role of proton based radiotherapy, which is being explored in the BN001 trial. Finally, with the advent of the MRLinac, it is necessary to determine the role it may play in the treatment of GBMs.

There are limited data regarding the rate of local progression during radiation therapy for GBM discussed below. MRLINAC provides excellent soft tissue imaging for image guided radiotherapy and the option of adaptive therapy, as well as enabling assessment of tumor response throughout treatment. This technology will also aid in the clinical decision-making process after a course of radiotherapy is complete which is particularly useful in patients with GBM. Essentially, the majority of patients with GBM will show disease progression at some point. Utilizing an MRLinac to detect progression or regression during radiotherapy will allow for radiotherapy field alteration and systemic therapy escalation if necessary (Figure 4).

Target dynamics during chemoradiation therapy has recently been examined for patients with GBM. A prospective study evaluated sequential MRI scans at multiple time points during chemoradiotherapy. Gross target volume (GTV) and clinical target volume (CTV) size, as well as tumor migration were evaluated at fraction 0, fraction 10, fraction 20, and 1 month after treatment. The majority of patients enrolled saw shrinkage of the gross tumor volume with a median volume decrease from 18.4 cm^3^ at fraction 0 to 14.7 cm^3^ at fraction 10, 13.7 cm^3^ at fraction 20, and 13.0 cm^3^ at one month after treatment. Tumor shrinkage was also associated with tumor migration with median GTV and (CTV) migrations being greater than 5 mm in 46% (54%) of patients at fraction 10, 50% (58%) of patients at fraction 20, and 52% (57%) of patients at one months after treatment [88]. These large tumor migrations during chemoradiotherapy expose patients to a potential geographic miss on subsequent boost volumes. These findings are not unique to this study as geographic miss due to tumor migration has been a concern noted in prior studies as well [89,90,91]. Consistent findings of tumor migration during treatment further reinforce the need to evaluate the role of magnetic resonance image guided radiotherapy (MR-IGRT) and on-table treatment plan adaptive radiotherapy for patients undergoing chemoradiotherapy for GBM.

A case series has explored daily MR-IGRT images of three GBM patients that were treated on an MRLINAC. Patients enrolled in this study were treated to the current standard of care with resection followed by fractionated radiotherapy to 60 Gy in 30 fractions with concurrent and adjuvant TMZ. With the MRLINAC on-board imaging capabilities, daily measurements of the resection cavity, associated edema, and T2-hyperintense residual tissue were obtained. There was a general trend of daily decrease in the resection cavity volume in the 3 patients analyzed. One patient initially had increased edema volume through the first 13 fractions, which then began to decrease until the end of treatment [92]. Given the gradual decrease noted in the cavity size and edema volume, regular interval imaging with MR-IGRT may allow for adaptive radiotherapy planning leading to reduced treatment fields and sparing of normal brain tissue. In other cases, where a brain tumor may grow during treatment, the inverse may be done and treatment field size may be increased. Finally, the authors of this case series also note that frequent imaging will also allow for monitoring of changes in cerebral edema which may allow for correlation of clinical symptoms and for monitoring of response to steroids [92].

A recent review by Maziero et al. featured early uses of the MRLINAC in following GBM during radiation therapy. The review featured a study by Jones et al. in which four of 14 patients who underwent daily imaging during their MR-guided radiation therapy demonstrated greater than 25% increases in the volume of T2 hyperintensity [93]. Three of the four patients experienced volume increase late in treatment. The review noted that in a study by Tsien et al., patients who later experienced progressive disease had significantly reduced rCBV during the third week of treatment [94]. Yang et al., also included in the review, showed tumors responding to RT showed increased ADC values during treatment [95,96].

The benefits of dose escalation through traditional radiotherapy techniques for patients with GBM have failed to yield a survival advantage. Dose escalation has been attempted in multiple Radiation Therapy Oncology Group (RTOG/NRG) trials over the past 5 decades. These trials explored multiple avenues of dose escalation such as using boost volumes, twice-daily fractionation, and hyperfractionation. The historical RTOG dose escalation trials failed to show improved overall survival. Furthermore, they showed greater toxicity with dose escalation [12,13,14,97]. While these prior dose escalation studies relied on older treatment techniques, a study out of the University of Michigan utilized a more modern approach and assessed dose escalation through 3D conformal radiation techniques. Multiple treatment arms were used with dose escalation ranging from 70–90 Gy. Although no survival benefit was detected, there was no significant increase in morbidity or mortality in patients receiving dose escalation through more modern radiotherapy techniques [98].

While older dose escalation studies have failed to show a survival benefit, dose escalation continues to be considered. More recently, with advances in MR spectroscopy (MRS), dose escalation for patients with GBM has been re-examined. In a pilot trial among 3 centers, MRS measuring levels of the brain metabolites choline (Cho) and N-acetylaspartate (NAA) were used to localize regions at high risk of recurrence as prior literature has shown that elevated Cho/NAA ratios confers a recurrence risk. The Cho/NAA ratio was normalized to contralateral normal appearing white matter. Radiotherapy plans were then developed by creating a GTV1 and GTV2 based on T2-FLAIR and T1 contrast enhanced MRI. This was followed by expanding each GTV by 5 mm to create CTV1/CTV2. Additionally, GTV3 (=CTV3) was created based on MRS findings and was generated by combining residual contrast enhancing tumor tissue and tissue with a Cho/NAA ratio greater than 2 times the normal appearing white matter. All clinical target volumes were expanded by 3 mm to create PTV1/PTV2/PTV3, which were treated to a prescribed dose of 50, 60, and 75 Gy, respectively. The median overall survival was 23.0 months. While there was an increased risk of radiation necrosis, it was clinically manageable and did not result in grade 3 or higher toxicity [99]. These promising findings and manageable toxicity may revive dose escalation that is guided by advanced imaging findings such as those detected on spectroscopic MRI.

## 11. Conclusions

Strides have been made in the treatment of GBM as described above; however, more work is needed to improve outcomes for patients with this disease. MRLINAC technology allows clinicians to leverage imaging information gathered during radiation therapy to adapt therapy for a patient while actively undergoing treatment. There is a significant need to understand how imaging changes may correlate to outcomes during treatment for GBM as these tumors have a poor prognosis and treatment tailored to the tumor characteristics may improve outcomes. Some of the advantages of MR guided therapy include facilitating a more detailed study of tumor and normal tissue response during chemo-radiation therapy, providing a mechanism to adapt therapy based on imaging changes, identifying new imaging biomarkers for tumor response as well as normal tissue response. These avenues could provide a more tangible way to evaluate pseudoprogression and radiation necrosis with radiogenomics as a mechanism to correlate imaging findings to genomic biomarkers. Multidisciplinary collaborations using MRLINAC based therapy are urgently needed to try and improve the dismal prognosis for patients with GBM.

## Figures and Tables

**Table 1:** mRANO Criteria.

Response vs. Progression	Change in Sum of Product Diameters	Change in Volumetric Measurement	New Measurable Lesion	Corticosteroids	Clinical Assessment
Complete Response	100% Decrease	100% Decrease	No	Off Corticosteroids or on Physiologic Replacement Dose	Stable or Improved
Partial Response	≥50% Decrease	≥65% Decrease	No	Corticosteroid Dose Is Same or Lower	Stable or Improved
Progressive Disease	≥25% Increase	≥40%Increase	Yes	NA	Worse and not attributable to other causes or change in steroid dose
Stable Disease	<50% Decrease to <25% Increase	<65% Decrease to <40% Increase	No	NA	

Measurable new lesion must measure at least 1 × 1 cm. Each response/progression is confirmed after 4 weeks. At the second scan after 4 weeks, the new measurable lesion is added to the sum of product diameters or volumetric measurement. Progression must measure at least 25% on both the first and second (after 4 weeks) scans. New lesion outside the radiation field indicates progressive disease. Modified from Ellingson BM, Wen PY, Cloughesy TF. Modified Criteria for Radiographic Response Assessment in Glioblastoma Clinical Trials. Neurotherapeutics 2017; 14:307-320.
